# Leveraging serology to titrate immunisation programme functionality for diphtheria in Madagascar

**DOI:** 10.1017/S0950268822000097

**Published:** 2022-01-13

**Authors:** Solohery L. Razafimahatratra, Arthur Menezes, Amy Wesolowski, Lala Rafetrarivony, Simon Cauchemez, Richter Razafindratsimandresy, Aina Harimanana, Tania Crucitti, Jean Marc Collard, C. J. E. Metcalf

**Affiliations:** 1 Immunology of Infectious Diseases Unit, Institut Pasteur de Madagascar, Antananarivo, Madagascar; 2 Experimental Bacteriology Unit, Institut Pasteur de Madagascar, Antananarivo, Madagascar; 3 Department of Ecology and Evolutionary Biology, Princeton University, Princeton, NJ, USA; 4 Department of Epidemiology, Johns Hopkins Bloomberg School of Public Health, Baltimore, MD, USA; 5 Mathematical Modelling of Infectious Diseases Unit, Institut Pasteur, UMR 2000, CNRS, Paris, France; 6 Virology Unit, Institut Pasteur de Madagascar, Antananarivo, Madagascar; 7 Epidemiology and Clinical Research Unit, Institut Pasteur de Madagascar, Antananarivo, Madagascar; 8 Center for Microbes, Development and Health (CMDH), Institut Pasteur of Shanghai/Chinese Academy of Sciences, Shanghai, China (Current Address); 9 Princeton School of Public and International Affairs, Princeton University, Princeton, NJ, USA

**Keywords:** Diphtheria, pertussis, serology, Madagascar

## Abstract

Diphtheria is a potentially devastating disease whose epidemiology remains poorly described in many settings, including Madagascar. Diphtheria vaccination is delivered in combination with pertussis and tetanus antigens and coverage of this vaccine is often used as a core measure of health system functioning. However, coverage is challenging to estimate due to the difficulty in translating numbers of doses delivered into numbers of children effectively immunised. Serology provides an alternative lens onto immunisation, but is complicated by challenges in discriminating between natural and vaccine-derived seropositivity. Here, we leverage known features of the serological profile of diphtheria to bound expectations for vaccine coverage for diphtheria, and further refine these using serology for pertussis. We measured diphtheria antibody titres in 185 children aged 6–11 months and 362 children aged 8–15 years and analysed them with pertussis antibody titres previously measured for each individual. Levels of diphtheria seronegativity varied among age groups (18.9% of children aged 6–11 months old and 11.3% of children aged 8–15 years old were seronegative) and also among the districts. We also find surprisingly elevated levels of individuals seropositive to diphtheria but not pertussis in the 6–11 month old age group suggesting that vaccination coverage or efficacy of the pertussis component of the DTP vaccine remains low or that natural infection of diphtheria may be playing a significant role in seropositivity in Madagascar.

## Introduction

In the pre-vaccine era, diphtheria was a leading cause of childhood mortality [1]. The etiological agent *Corynebacterium diphtheriae* (or, more rarely, *Corynebacterium ulcerans*) causes either respiratory or cutaneous disease, with the former associated with a higher risk of mortality, and reportable to the World Health Organization (WHO). In untreated unvaccinated individuals, the case fatality rate (CFR) may be around 29%, but health care improvements have driven this number close to zero if patients are properly diagnosed [2]. This progress has occurred alongside notable reductions in case numbers as a result of the expansion of immunisation by vaccination beginning after World War II [1]. Nevertheless, the CFR continues to be as high as 33% in resource-poor settings [2, 3]. Vaccination coverage is incomplete in many parts of the world [4] and diphtheria outbreaks can have a devastating impact, particularly in crisis situations, with a recent example amongst refugees from Myanmar [5]. Even in highly vaccinated populations where the level of indirect protection should be high, unvaccinated individuals may be vulnerable following the introduction of the pathogen [6–8].

**Fig. 1.**
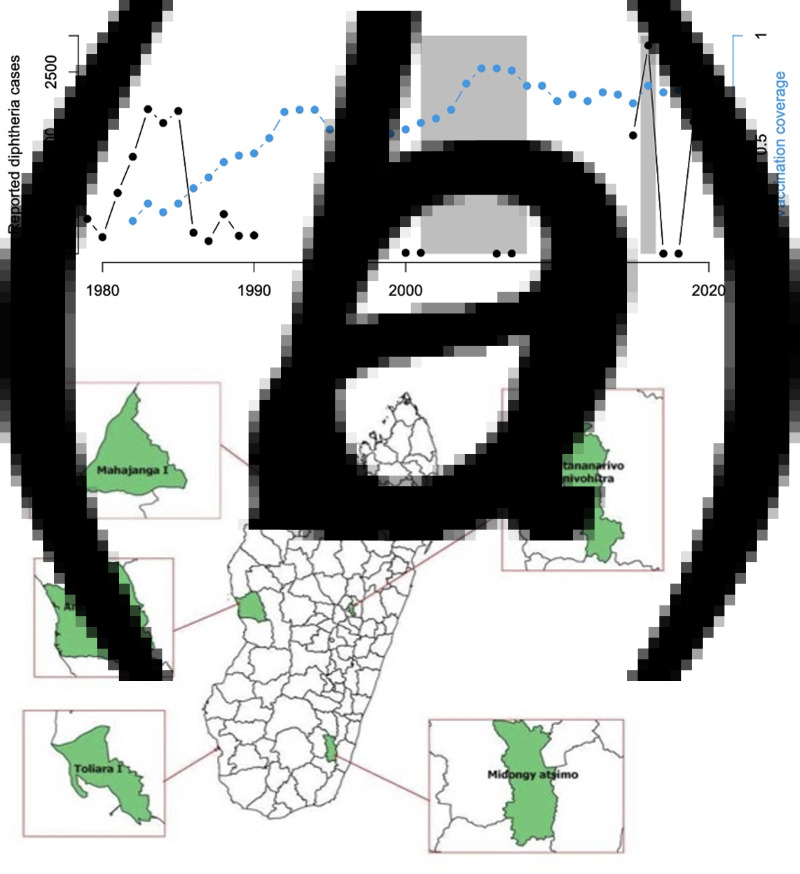
Diphtheria in Madagascar (a) Time course of reported cases (black) and reported vaccination coverage (blue) from the WHO Global Health Observatory showing the range of years during which children who were 8–15 years old in 2016 would have been vaccinated (left grey polygon) and that children aged 6–11 months old would have been vaccinated (right grey polygon); (b) Map of Madagascar and location of the five study sites, Antananarivo Renivohitra (high density urban setting with 1 274 225 inhabitants), Antsalova (low density rural setting with 60 000 inhabitants), Midongy Atsimo (low density rural setting with 49 000 inhabitants), Mahajanga I (urban setting with 246 000 inhabitants) and Toliara I (urban setting with 168 700 inhabitants).

Other than reported cases, relatively little is known about the epidemiology of diphtheria in the Indian Ocean Region [9], and specifically in Madagascar. From 2011 to 2015, Madagascar reported the third-largest number of diphtheria cases per country (1633 cases) after India (18 350 cases) and Indonesia (3203 cases), despite decades of distribution of the diphtheria-tetanus-pertussis (DTP) vaccine (Fig. 1a) [10]. Diphtheria vaccines were adopted in Madagascar in 1976 as part of the Expanded Programme on Immunisation [11]. Currently, in Madagascar, three doses of the DTP, hepatitis B and *Haemophilus influenzae* B (DTP-HepB-Hib) vaccine are administered at 6, 10 and 14 weeks of age, following the WHO recommended vaccination schedule [11, 12]. Booster shots are recommended but not administered in Madagascar. In 2016, coverage for all three doses was estimated to be relatively high, especially on the Central High plateau, but these estimates were associated with considerable uncertainty [4], and there was also clear spatial variability.

**Fig. 2.**
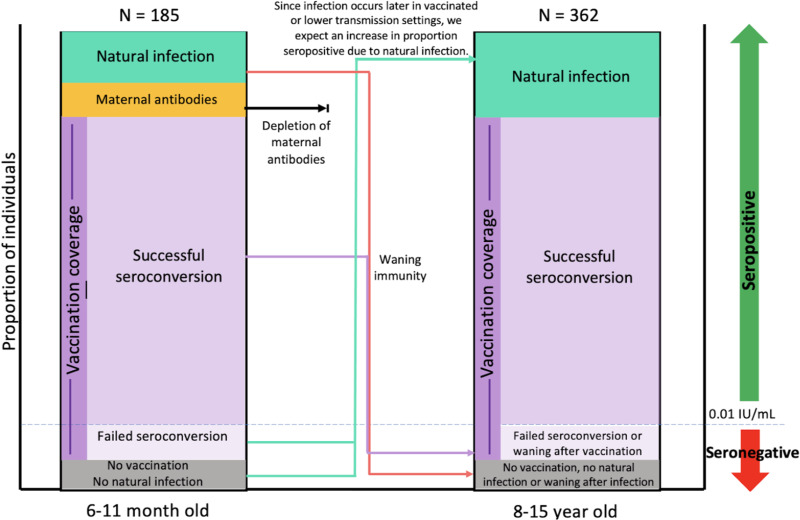
Expected mechanisms responsible for diphtheria seropositivity or lack thereof among both age groups. Among the 6–11 month olds, we expect the largest proportion of seropositivity (IU/ml⩾0.01) to be attributed to vaccination with smaller proportions of seropositivity attributed to natural infection (since it is unlikely that children will be infected so rapidly) and the presence of maternal antibodies. Given complete diphtheria vaccine seroconversion efficacy, we expect 3% of vaccinated individuals to fail to seroconvert. Lastly, we expect some proportion of seronegative individuals (IU/ml < 0.01) to be attributed to no vaccination and no natural infection. Similarly, we expect the largest proportion of seropositivity (IU/ml⩾0.01) to be attributed to vaccination in the 8–15 year old group. Maternal antibodies no longer play a role in this group as they have fully waned while the proportion of seropositivity due to natural infection is expected to increase since individuals have had more time to become infected. Lastly, we expect some proportion of seronegative individuals (IU/ml < 0.01) to be attributed to incomplete or no vaccination, no natural infection or antibody waning following vaccination or natural infection.

Uncertainties about vaccine coverage alongside high reported case numbers, and considerable potential for underreporting of infections due to incomplete surveillance make characterising the burden of this pathogen and the effectiveness of the vaccination programme in Madagascar an important public health question. One approach to probing the epidemiology of diphtheria is via serology, or measurement of antibody titres [13], which generally increase following infection or vaccination. Serology has the potential to provide a more complete picture than case counts in identifying the footprint of diphtheria, as a recent review suggests many infections may be asymptomatic [2] and thus unlikely to be captured by classic surveillance. However, interpreting the signature of serology is complicated as immunity from infection and vaccination cannot be distinguished [14]. Furthermore, diphtheria antibodies wane over time [15], so that seronegative individual may still have been vaccinated or infected. The epidemiology of the infection and the context of immunisation can help inform interpretation. These theoretical processes allow us to map out possible trajectories of seropositivity in the early years of life as a function of epidemiological and vaccinal context (Fig. 2). Given the reported high levels of DTP vaccination coverage and the number of reported diphtheria cases, we expected a large proportion of individuals in both age groups to be seropositive to diphtheria. Maternal antibodies might contribute to seropositivity in the 6–11 month old (mo) age group [16] and waning immunity is likely to contribute to seronegativity in the 8–15 year old age group [15].

Diphtheria seroprevalence has not previously been evaluated in Madagascar leaving a gap in understanding the landscape of immunity. Since the pertussis and diphtheria vaccines were combined in the mid-1940s [17], and are in use in combined form in Madagascar, interpretation of diphtheria serology titres can be strengthened by also considering serological status to pertussis. Here, we tested available Madagascar sera collected during a serology study on poliomyelitis in 2016 [18] and analysed in a pertussis serology study in 2020 [19] for anti-diphtheria IgG antibody levels to evaluate the immune profiles of children aged 6–11 months old and 8–15 years old, DTP vaccination coverage and diphtheria footprint in selected districts in Madagascar (Fig. 1b).

## Methods

### Study sample

A total of 593 sera of children aged 6 to 11 months from three districts and 8 to 15 years from five districts (Fig. 1b) collected May to September 2016 and analysed in the previous poliomyelitis and pertussis serology study [18, 19] were selected. With 12 sera depleted, and 34 lost, 547 were available for analysis (Table 1). Participants provided written consent and agreed that their serum could be used for the purpose of other infectious disease research. Possession of a vaccination card and nutritional status of children were also recorded when available (Supplementary Text S1).

**Table 1.** Characteristics of the study population

	No. of Children by Age groups (Percentage)	
	6–11 m	8–15 y
Total *N* = 547	185	362
Sex		
Male (*N* = 249)	90 (49%)	159 (44%)
Female (*N* = 298)	95 (51%)	203 (56%)
Vaccination card		
Present (*N* = 128)	123 (66%)	5 (1%)
Absent (*N* = 419)	62 (34%)	357 (99%)
Nutritional status		
Moderate Acute Malnutrition (*N* = 92)	32 (17%)	60 (17%)
Severe Acute Malnutrition (*N* = 118)	21 (12%)	97 (27%)
Normally Nourished (*N* = 326)	130 (70%)	196 (54%)
Outlier (*N* = 11)	2 (1%)	9 (2%)
Samples collection districts		
ANT (*N* = 125)	51 (28%)	74 (20%)
ATS (*N* = 116)	49 (26%)	67 (19%)
MAH (*N* = 81)	–	81 (22%)
MID (*N* = 149)	85 (46%)	64 (18%)
TOL (*N* = 76)	–	76 (21%)

m, months; y, years; ANT, Antananarivo Renivohitra; ATS, Antsalova; MAH, Mahajanga I; MID, Midongy Atsimo; TOL, Toliara I.

### Serological assay

Diphtheria toxoid IgG-specific antibody levels were determined using a commercial Anti-Diphtheria Toxoid IgG ELISA (Euroimmun, Germany) identified as a reliable anti-diphtheria IgG assay [20] (Supplementary Text S2). Individuals with diphtheria antibody levels under 0.01 IU/ml are highly susceptible to the disease while individuals with higher levels are associated with less severe symptoms [21–24]. Individuals with diphtheria antibody levels of 0.01 IU/ml have the lowest antibody titre which provides some degree of protection while individuals with ≥0.1 IU/ml are associated with long term protection [25]. Levels in between 0.01 and 0.09 IU/ml are considered to provide basic levels of protection against disease [26]. We used antibody titre levels at ≥0.01 IU/ml as the seropositivity threshold (sensitivity and specificity of ~94% [27]). For pertussis, the concentration of anti-PT IgG was determined using a commercially available ELISA kit (EUROIMMUN, Lübeck, Germany), see [19] for details; we set the lower limit of detection, 5 IU/ml, as the threshold for seropositivity. We opted to set a lower threshold to increase sensitivity and provide conservative bounds on vaccination.

### Statistical analysis

#### Analysis of diphtheria serological data

To characterise the landscape of diphtheria immunity, initially considering the diphtheria data alone, we focused first on the 6–11 mo, and characterised the lower bound on the proportion of unvaccinated children (defined by IU/ml <0.01) by the district using a generalised linear model with district fitted as a factor. We also evaluated the distribution of log antibody titres in this age group among those above the threshold for protection (IU/ml ≥ 0.01) using a linear model with nutritional status fitted as a factor to explore whether vaccinated (or possibly previously infected) individuals might have lower antibody titres if malnourished.

Among the 8–15 year olds, we also broadly characterised the proportion seronegative, using a generalised linear model with the district as a factor, noting that these children could have been vaccinated with the subsequent waning of immunity (although that would be very rapid since protective immunity to diphtheria is expected to take over 40 years to be lost [15]); or been infected with the subsequent waning of immunity, although again this is unlikely given the slow rate of waning. We also evaluated the distribution of log antibody titres in this age group among those above the threshold for protection (IU/ml ≥ 0.01) using a linear model to evaluate the impact of the district, as these could capture overall differences in transmission context. We did not include nutritional status in our analysis of 8–15 year olds, since nutritional status close to the age of vaccination is likely to be more relevant.

#### Analysis of combined diphtheria and pertussis serological data

To further probe the bounds on vaccination coverage, focusing on the 6–11 mo, we calculated the proportion of children who were seropositive for both pertussis (defined by IU/ml ≥ 5) and diphtheria (defined by IU/ml ≥ 0.01), one or the other, or neither; and estimated confidence intervals based on binomial uncertainty. We compared these proportions with the administrative coverage data (i.e., numbers of vaccine doses delivered divided by target population size [28]) available for the focal districts in the years 2015–2017 from the Direction of the Expanded Programme on Immunisation in Madagascar accessed on 07/13/2021. Vaccinated individuals who are young enough that their vaccinal immunity has yet to wane below the seropositivity threshold (aged less than 4–12 years for pertussis [29, 30], and less than 40 for diphtheria [15]), should predominantly have vaccine-derived antibodies to both diseases, while individuals with only antibodies to one may have experienced natural infection rather than complete vaccination, received antibodies from their mother or experienced failed seroconversion to one and not the other. Diphtheria and pertussis maternal antibodies vary a few months in their longevity and therefore cannot be ruled out as a cause of seropositivity in children in the 6–11 mo age group [16, 31, 32]. The probability of these scenarios depends on vaccine efficacy, the risk of infection, and the probability of seroconversion following natural infection.

## Results

### Diphtheria seropositivity

**Fig. 3.**
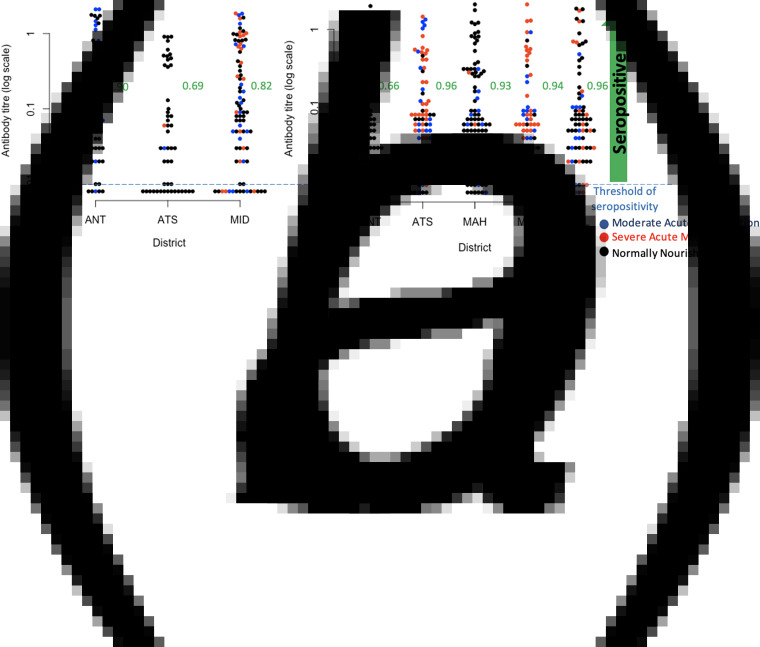
Observed diphtheria antibody titre concentrations. (a) Diphtheria antibody titre concentrations in children aged 6–11 mo across three districts for which data was available, colours indicate nutritional status, and proportions seropositive for diphtheria in the three districts as ordered on the figure are, respectively 0.90, 0.69, 0.82; (b) Diphtheria antibody titre concentrations in children aged 8–15 years across all five districts (colours show nutritional status as for 6–11 mo for comparison), and proportions seropositive for diphtheria are 0.66, 0.96, 0.93, 0.94, 0.965 across the districts as shown on the figure. Districts are: ANT, Antananarivo Renivohitra; ATS, Antsalova; MAH, Mahajanga I; MID, Midongy Atsimo; TOL, Toliara I.

Among the 185 children aged 6–11 mo, we found that there is a substantial proportion lacking diphtheria antibody titres suggesting that they had not been vaccinated or not seroconverted following vaccination (IU/ml < 0.01) (Fig. 3a). Overall, assuming the risk of infection is low, and assuming that seroconversion maps to immunity, 18.9% of children were not immunised to diphtheria by vaccination in the three districts where data were available (Fig. 3a), and with the odds of not being immunised by vaccination significantly higher in Antsalova than Antananarivo (Odds Ratio of 1.23, 95% confidence interval 1.05–1.43) but not in Midongy Atsimo (Odds Ratio of 1.08, 95% confidence interval 0.94–1.23). Based on the linear regression, the mean log antibody titre among the 148 6–11 mo children above the threshold for protection was significantly lower in children categorised as normally nourished (−0.75, 95% confidence interval −0.23 to −1.28) relative to the mean of −1.35, 95% confidence interval of −0.92 to −1.79, but an interpretation of this is unclear. This pattern remains if you restrict the analysis to 6–11 mo children seropositive to both diphtheria and pertussis who are likely to have been vaccinated.

Among the 362 children aged 8–15 years, we found a considerable proportion of children in Antananarivo (34% or 25 out of 74) showing no evidence of seroconversion by vaccination or infection (IU/ml < 0.01, Fig. 3b); the odds of being seronegative are significantly lower in the other districts (Odds Ratio of 0.74 in Antsalova, 95% confidence interval 0.67–0.82; Odds Ratio of 0.76 in Mahajanga, 95% confidence interval 0.69–0.84; Odds Ratio of 0.75 in Midongy Atsimo, 95% confidence interval 0.68–0.83; Odds Ratio of 0.74 in Toliara I, 95% confidence interval 0.67–0.81). Among the 321 children above the threshold for seropositivity, the mean log antibody titres is −2.90 in Antananarivo (95% confidence interval −3.28 to −2.25) with significantly higher titres in Antsalova (increased by 0.67, 95% confidence interval 0.16 to 1.18), Mahajanga (increased by 0.69, 95% confidence interval 0.20 to 1.18) and Toliara I (increased by 0.50, 95% confidence interval 0.01 to 0.99) but not Midongy Atsimo (0.45 with 95% confidence interval −0.05 to 0.97); however, this model only explains 2% of the variance, suggesting that there are many other sources of variability beyond regional effects. We also found that vaccination card possession did not reduce the unexplained variance in antibody titres for either 6–11 mo or 8–15 year olds, so we did not investigate this covariate further.

### Diphtheria and pertussis seropositivity

The efficacy of the diphtheria vaccine, or the probability that a fully vaccinated individual will seroconvert, is estimated to be ~0.97 [33, 34]. While the efficacy of the whole-cell pertussis vaccine, which is given in Madagascar, against disease is reported as being around ~78% [35], this need not map to seroconversion, as no clear serological correlate of protection has been found [12]. However, combined evidence of the magnitude of short term efficacy against disease [36] and presence of pertussis antibodies 1 month after the final dose of DTP vaccination [37] suggests that values of seroconversion after complete three dose vaccination of around 94% may be expected, and this is the quantity that we use. Among fully vaccinated children who have not been naturally exposed (and assuming that there is no individual variation in response to vaccination that would cause non-independence among responses to the different antigens), assuming the 94% cited for pertussis above, and 97% for diphtheria, we thus expect 91.2% to be seroconverted to both infections, with 0.2% seroconverted to neither, and 5.8% seroconverted to diphtheria but not pertussis, while 2.8% are seroconverted to pertussis but not diphtheria. Qualitative patterns are unchanged across the reported range of seroconversion probabilities following complete vaccination. Deviations from these patterns will provide information as to missed vaccination and risk of infection or presence of maternal antibodies. Deviations could also be attributed to differential seroconversion efficacies in individuals with incomplete DTP vaccination, discussed below. We assumed that the probability of seroconversion following natural infection is high [14].

Among the 185 children aged 6–11 mo, 64 (34.6%, 95% CI 0.281–0.417) were seropositive for both pertussis and diphtheria and 33 (17.8%, 95% CI 0.130–0.240) children were seropositive for neither, while two children (1.1%, 95% CI 0.002–0.004) were seropositive for pertussis only and 86 children (46.5%, 95% CI 0.394–0.537) were seropositive for diphtheria only (Table 2). In a three dose fully vaccinated population of 185 children, we expect 169 (91.1%) individuals seroconvert to both pertussis and diphtheria while 5 (2.8%) individuals seroconvert to pertussis only and 11 (5.8%) individuals seroconvert to diphtheria only. We also expect that 0 (0.18%) individuals will fail to seroconvert to either. It is also possible that differential seroconversion efficacies in individuals with incomplete DTP vaccination might be at play.

**Table 2.** Diphtheria and pertussis seropositivity and estimated vaccination coverage among children aged 6–11 months old. Diphtheria (DP) and pertussis (PT) seropositivity in children aged 6–11 months in the three districts for which data was available; excess number of diphtheria only seropositive individuals; expectations given vaccine efficacy under complete vaccination and estimated vaccination coverage

	6–11 month old			
Seropositive	PT & DP	PT	DP	Neither
ANT (*N* = 51)	23 (45.1%)	2 (3.9%)	23 (45.1%)	3 (5.9%)
ATS (*N* = 49)	11 (22.4%)	0	23 (46.9%)	15 (30.6%)
MID (*N* = 85)	30 (35.3%)	0	40 (47.1%)	15 (17.6%)
Total (*N* = 185)	64 (34.6%)	2 (1.1%)	86 (46.5%)	33 (17.8%)
Expected under complete vaccination (*N* = 185)	169 (91.1%)	5 (2.8%)	11 (5.8%)	0 (0.18%)
Expected seropositivity under estimated vaccination coverage				
Bound 1: 37.9%	64 (34.6%)	2 (1.1%)	4 (2.2%)	115 (62.2%)
Difference	0	0	82 (44.3%)	−82 (−44.3%)
Bound 2: 34.6%	58 (31.4%)	2 (1.1%)	4 (2.2%)	121 (65.4%)
Difference	6 (3.2%)	0	82 (44.3%)	−88 (−47.6%)

ANT, Antananarivo Renivohitra; ATS, Antsalova; MID, Midongy Atsimo.

The vaccine seroconversion rates for complete diphtheria and pertussis vaccination allow us to estimate the vaccination coverage expected given the observed data on seroconversion to both infections. To bound vaccination coverage, we can first assume that all individuals seropositive for pertussis and diphtheria are fully vaccinated, which equates to making the simplifying assumption that individuals who are seronegative for either (DP or PT) or both were not fully vaccinated (bound 1). Given that 64 (34.6%) individuals aged 6–11 mo were seropositive for both, and the probabilities of seroconversion, this suggests vaccination coverage among the 6–11 mo is 37.9%. With a 37.9% vaccination rate, we would expect two individuals seropositive for pertussis only and four individuals seropositive for diphtheria only. We found two individuals seropositive for pertussis only and 86 individuals seropositive for diphtheria only; an excess of 82 diphtheria only seropositive individuals, suggesting either the high incidence of diphtheria, the presence of natural passive immunity or high levels of failed seroconversion to the pertussis component of the DTP vaccine potentially via incomplete vaccination (<3 doses) (Table 2).

To provide an alternate bound on complete vaccination coverage, we can assume that all individuals seronegative for either pertussis, or diphtheria, or both are not fully vaccinated, which equates to making the simplifying assumption that individuals who are seronegative for one of the two (DP or PT) were not fully vaccinated, rather than failed to seroconvert (bound 2). Given that 33 (17.8%) individuals aged 6–11 months were seronegative for both, 2 (1.1%) were seronegative for diphtheria and 86 (46.5%) were seronegative for pertussis, we predict that vaccination coverage among the 6–11 mo is 34.6% (bound 2). With a 34.6% vaccination rate, we would expect two individuals seropositive for pertussis only and four individuals seropositive for diphtheria only. We found two individuals seropositive for pertussis only and 86 individuals seropositive for diphtheria only; an excess of 82 diphtheria only seropositive individuals, also suggesting a high incidence of diphtheria, natural passive immunity or high levels of failed seroconversion to the pertussis component of the DTP vaccine potentially via incomplete vaccination (<3 doses) (Table 2).

**Fig. 4.**
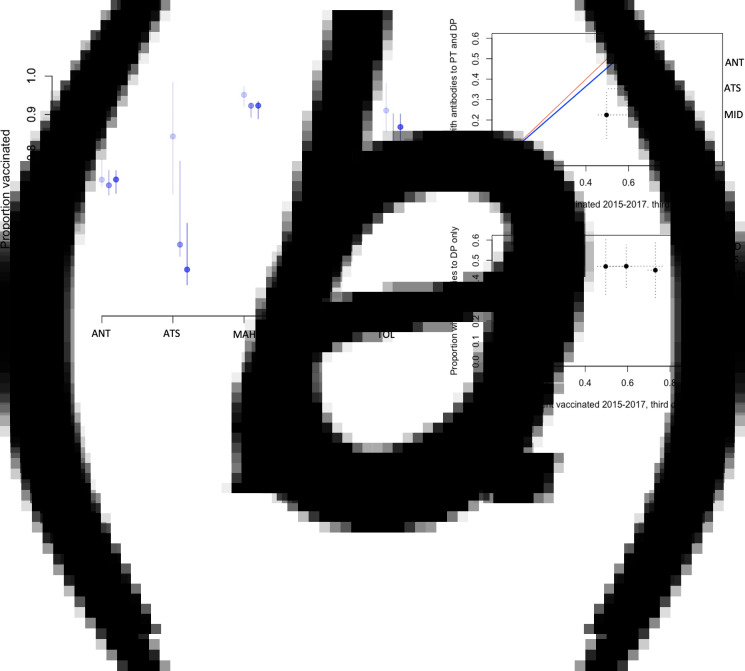
Comparing estimates of vaccination coverage and antibody titres for individuals 6–11 months old. (a) Administrative coverage data (i.e., numbers of vaccine doses delivered divided by target population size) in each of the 5 districts across 2015–2017 (y axis) showing the median (filled point across 2015, 2016, and 2017) and the range (lines, lowest and highest in 2015, 2016, 2017) for the first, second and third dose, in order, darker blue indicates the third dose. (b) Median and range of administrative coverage data for 2015, 2016, and 2017 (*x* axis) plotted against the proportion seropositive for both DP and PT in each district (*y* axis) also showing for comparison the *y* = *x* line in red, and the expectation of number of individuals seropositive for both DP and PT if the proportion estimated as vaccinated by administrative coverage (x axis) had been reached and correcting for seroconversion (blue line *y* = 0.91x). The distance between the blue line and the points indicates one measure of shortfalls in vaccination. (c) Same *x*-axis as Panel B plotted against the proportion in each district seropositive for DP only (*y* axis). Districts are: ANT, Antananarivo Renivohitra; ATS, Antsalova; MAH, Mahajanga I; MID, Midongy Atsimo; TOL,Toliara I.

We further probed this dataset to explore the degree to which serological data on diphtheria and pertussis might suggest different complete vaccination coverage amongst the districts, with a particular focus on numbers of diphtheria only seropositive children in each district, potentially indicating higher rates of natural infection (although a failure of the pertussis component of the vaccine is also possible). We found that although the estimated vaccination coverage bounds vary among the three districts (Antananarivo Renivohitra (45.1%–49.5%), Midongy Atsimo (35.3%–38.7%), Antsalova(22.4%–24.6%)), the number of diphtheria only seropositive children appears to be at a comparable level (Supplementary Table S1, Fig. 4c). Reported administrative coverage levels for complete 3 dose vaccination in 2015–2016 averaged 74% for Antananarivo Renivohitra, 63.5% for Midongy Atsimo and 48% for Antsalova. We did not conduct this analysis for the 8–15 year olds since the interpretation of seropositivity is complicated by antibody waning and boosting events (Supplementary Table S2).

Finally, we compared seropositivity to the administrative coverage data (i.e., numbers of vaccine doses delivered divided by target population size) in our focal districts. Administrative coverage fluctuates considerably through time (Fig. 4a). Given likely uncertainty in translation between administrative coverage and vaccine doses delivered [38], we take the median and the range across the 3 years available, and plot the median against the proportion of individuals seropositive for both pertussis and diphtheria, to compare this alternative window onto the proportion of individuals vaccinated (Fig. 4b). Both measures are uncertain, but the positive relationship between the two across districts suggests that they may be capturing the broader phenomenon of vaccination coverage. This comparison also suggests potential overestimation of children successfully immunised provided by administrative measures of coverage. We also take the median and the range across the 3 years available, and plot it against the proportion of individuals seropositive for diphtheria only, to investigate the impact of vaccination coverage on DP only seropositive individuals which are believed to either be attributed to natural infection, passive natural immunity or failed seroconversion to PT following partial DTP vaccination (Fig. 4c). The similar number of individuals seropositive for DP only could either indicate a comparable level of circulating diphtheria in all 3 districts despite varying levels of vaccination coverage, the presence of vaccine acquired maternal antibodies or similar levels of failed vaccine-induced seroconversion to pertussis (Fig. 4c, Supplementary Table S1).

It is important to note that there is no universal threshold for pertussis seropositivity. Other pertussis serology studies use 40 IU/ml as a cutoff for seropositivity instead of the 5 IU/ml used here [39–41]. If we implement this higher cutoff, 222 individuals categorised as seropositive to pertussis move into the seronegative category. This consequently increases the proportion of individuals seropositive to diphtheria only from 192 to 414 (35.1% to 75.7%) indicating either a higher presence of circulating diphtheria or lower efficacy of the pertussis component of the vaccine than we propose here. In Supplementary Table S3 and S4, we display updated versions of Table 2 and Supplementary Table S2 with an adjusted pertussis seropositive threshold of 40 IU/ml. Estimated vaccination coverage among the 6–11 mo individuals decreased from 34.6%–37.9% to 4.9%–9.9% (Supplementary Table S3). Ultimately, we set 5 IU/ml, the lower limit of ELISA detection, as the pertussis seropositive cutoff in order to provide a conservative upper bound of individuals likely to have been infected or vaccinated by opting for higher sensitivity. This allowed us to capture individuals who may have experienced rapid waning or low antibody production following infection or vaccination.

## Discussion

Understanding gaps in immunity and shortfalls in vaccination coverage for vaccine-preventable infections is a key question in public health. Here, we measured serological data on diphtheria and leveraged it with previously acquired pertussis serological data to expand our understanding of the characteristics of the landscape of immunity, the footprint of diphtheria and DTP vaccination coverage in Madagascar. Since the DTP vaccine is among the earliest delivered, it is often considered a good proxy for health system functioning and has recently been associated with less severe COVID-19 [42]. These results therefore provide a broader window onto the functionality of the expanded programme on immunisation in Madagascar.

First, since the vaccination series for diphtheria should be completed by 14 weeks, the proportion of children aged greater than 14 weeks that have no antibodies provides a lower bound on the number of children who have not been reached by the vaccination programme - as long as these children are young enough to ensure that seropositivity has not been lost [43, 44]. As diphtheria antibodies have an estimated half-life of 19 years [45], focusing on children aged less than 1 year makes loss of seropositivity by waning unlikely. If most vaccinated children seroconvert, the proportion of diphtheria seropositive children aged 6–11 mo will provide an upper bound on vaccinated individuals (and thus a conservative lower bound on unvaccinated individuals), as there is still the possibility that some children are seropositive due to natural infection or the presence of maternal antibodies. Our analysis suggests both shortfalls and spatial heterogeneity in vaccination coverage, via several lines of evidence, and provides insight into the prevalence of zero-dose children. First, a large proportion of children remained seronegative for diphtheria antibodies at 6–11 months (between 10 and 31%), an age too young to have experienced waning of immunity. This proportion varies from district to district (Fig. 3a). Second, we also find strikingly large proportions (between 4 and 34%) of diphtheria antibody seronegativity in older children aged 8–15 years (Fig. 3b). The latter observations are harder to interpret as both natural infection and waning of immunity might have occurred within this age group, which also experienced variable levels of vaccination coverage (Fig. 1a, Fig. 2). Among the older age group, the highest proportion of diphtheria antibody seronegative children is found in Antananarivo, where vaccination coverage is expected to be the most elevated [19]. This suggests that natural infection may have contributed to seroconversion in the other regions, although differential antibody waning may also have played a role.

Second, an important piece of the efficacy of immunisation programmes is the individual heterogeneity in antibody titres achieved in response to vaccination [15]. Describing this variation and identifying the underlying biological drivers remain important open questions. Malnutrition has been shown to reduce the acquisition of immunity to some vaccines [46]. Although previous work indicates little evidence for this in diphtheria [47], a recent birth cohort study found that haemoglobin increased anti-diphtheria IgG, at week 24, and anaemia reduced sero-conversion [48], an argument for iron supplementation at the time of vaccination. Overall, this suggests that indicators of malnutrition might thus provide an additional window onto a determinant of immunisation by vaccination to diphtheria. Based on this, in children less than 1 year who do show an antibody response (and are thus likely to have been vaccinated), we would expect lower antibody titres in children classified as malnourished relative to those not. Lower antibody titres among malnourished children did not emerge as a significant variable among the youngest age group. This pattern is hard to interpret given the current range of data (for example, malnourished children might experience less vaccine-induced protection, and thus be more subject to infection that would boost titres, but one cannot be sure based on the data available). One feature that very clearly emerges is the broad heterogeneity in antibody levels detected which could be a result of boosting events due to circulating diphtheria, antibody decay, heterogeneity in a number of doses received and individual heterogeneity in immune response (Fig. 3).

Since the diphtheria and pertussis vaccines are combined, children who are seropositive for both (or seronegative for both) provide an alternative lens onto the range of vaccination coverage and the prevalence of zero-dose children while children who are seropositive to just one can provide insight into either the levels of circulating disease, presence of maternal antibodies (for the younger age group), or failed seroconversion to either pertussis or diphtheria. We approached this from two angles: assuming that seronegativity for both or just one reflects the absence of vaccination, or assuming that joint seropositivity reflects the presence of vaccination. Both approaches indicated that vaccination coverage is significantly lower than reported and that there is likely to be either considerable circulation of diphtheria within Madagascar, vaccine acquired maternal antibodies or substantial levels of failed seroconversion for pertussis after vaccination (Table 2, Supplementary Table S1, Fig. 4b c). Although vaccination coverage varied among the three districts (Antananarivo Renivohitra (45.1%–49.5%), Midongy Atsimo (35.3%–38.7%), Antsalova(22.4%–24.6%)), the proportion of excess diphtheria only seropositive individuals was rather similar (43.1%–44.9%) suggestive of either similar levels of circulating diphtheria (1) infecting infants or (2) their mothers or (3) similar levels of vaccine acquired maternal antibodies being passed down (Supplementary Table S1). Possibilities (1) and (2) are supported by a recent systematic review suggesting that vaccination has little effect on preventing infection since those with asymptomatic infections still transmit, but at only 24% of the rate of symptomatic cases [2]. This pattern suggests that the transmission will be harder to control with vaccination than originally believed and that antibiotics and isolation are critical for an outbreak response. Possibility (1) would signify a lower age of diphtheria infection which is alarming considering children aged less than 5 are more likely to die from symptomatic infection than adults aged 20 and over (relative risk = 1.5) [2]. Better surveillance of diphtheria cases is needed to shed light on the true incidence within Madagascar. Possibilities (2) and (3) suggest that the 6–11 mo individuals seropositive for diphtheria only have missed their vaccination, and will soon lose their maternal antibodies increasing their likelihood of experiencing a more severe diphtheria infection (case fatality ratio of untreated, unvaccinated cases is 29%) [2]. This is alarming as it can lead to an undercount of the number of zero-dose children in Madagascar. All three possibilities are evidence of causes for concern and demonstrate the need for a better understanding of diphtheria's transmission and epidemiology in Madagascar and the implications for vaccination timelines. Upon revisiting our expectations laid out in Figure 2 with the serology data from the 6–11 month old individuals, we see that the overall proportion of individuals seropositive for diphtheria is likely lower in this age class with overall vaccination coverage contributing less to seropositivity, and natural infections or maternal antibodies contributing more than we had originally expected.

Although the literature suggests that three dose vaccine seroconversion efficacy for diphtheria and pertussis is at comparable high levels, it is possible that the seroconversion efficacy for 1–2 doses of DTP vaccine is higher for diphtheria than for pertussis. For example, it might take three doses of DTP for individuals to seroconvert to pertussis but only 1–2 doses for diphtheria seroconversion. Therefore this could be another explanation for the large number of individuals seropositive for diphtheria only which implies that incomplete DTP vaccination series is prevalent in Madagascar (Fig. 4a) and of more concern for pertussis than diphtheria. Similarly, a recent study measuring diphtheria and pertussis IgG levels in 484 children in China found lower antibody levels and protection to pertussis alone [39]. Researchers speculated that this result might be due to a suboptimal immune response to the pertussis component of the DTP vaccine. Consequently, the high proportion of diphtheria only seropositive individuals shown in our study either suggests high levels of circulating diphtheria, incomplete/missing vaccination or suboptimal immune response to the pertussis component of the vaccine.

Selection bias may be present since only individuals with available health centre registries were included excluding those not in the registries who are likely under-vaccinated [18, 19]. Our analysis is also limited by the small number of districts and samples reducing the generalisability of our results. Lastly, since IgG antibodies are only one defence mechanism used by the immune system to fight off infection, measuring memory B cells and memory T cells would provide a more complete picture of the landscape of immunity.

By leveraging serological and administrative vaccination coverage data, alongside knowledge of the mechanisms of diphtheria and pertussis antibody dynamics, we provided an approach to unpick the landscape of immunity, examine the footprint of diphtheria and bound estimates of immunisation by vaccination. This approach indicates variability in vaccination coverage in Madagascar, and potential ongoing circulation of diphtheria or suboptimal immune response to the pertussis component of the DTP vaccine. It also indicates the potential of serological approaches for better characterising the landscape of immunity, a key question in public health, especially in the context of vaccine-preventable infections [49].

## Supplementary material

For supplementary material accompanying this paper visit http://doi.org/10.1017/S0950268822000097. click here to view supplementary material

## Data

Data and code are available at: https://github.com/AMenezes97/Diphtheria_Madagascar
