# Supplementary material for: Leveraging serology to titrate immunisation programme functionality for diphtheria in Madagascar
Source: Epidemiol Infect. 2022 Jan 13;150:e39. doi: 10.1017/S0950268822000097 (PMC8888278; doi:10.1017/S0950268822000097)
Supplement: Supplementary file 1 [file hygsup.zip › S0950268822000097sup002.docx]

**Epidemiology and Infection**

**Leveraging serology to titrate immunization program functionality for diphtheria in Madagascar**

Solohery L Razafimahatratra, Arthur Menezes, Amy Wesolowski, Lala Rafetrarivony, Simon Cauchemez, Richter Razafindratsimandresy, Aina Harimanana, Tania Crucitti, Jean Marc Collard, CJE Metcalf

**Supplementary Material**

**Supplementary Text**

**Methods**

**Supplementary Text S1. Study Sample**

Possession of a vaccination card and the associated data was recorded when available. The nutritional status of the children was defined based on a Z-score of weight-for-height [[46]](https://paperpile.com/c/vuVPt1/wj0M) with moderate acute malnutrition (MAM) corresponding to a Z-score between -3 and −2 and severe acute malnutrition (SAM) corresponding to a Z-score below −3. Children normally nourished (NN) were defined based on a Z-score between -2 and 2. Measurements of Z-scores greater than or equal to 6 and less than or equal to -6 were discarded as likely measurement error.

**Supplementary Text S2. Serological assay**

We used a commercial Anti-Diphtheria Toxoid IgG ELISA (Euroimmun, Germany) to measure diphtheria toxoid IgG-specific antibody levels. The reagent kit contains four ready-to-use calibrators - Calibrator 1 (2 International Units per Milliliter (IU/mL)), Calibrator 2 (1 IU/mL), Calibrator 3 (0.1 IU/mL), Calibrator 4 (0.01 IU/mL) for the determination of the IgG concentration and two control sera (one positive and one negative) for run validation. For the construction of the calibration curve, the optical density (OD) of each calibrator (y-axis, linear) was plotted against its concentration (x-axis, logarithmic). The sera were tested in a 1:101 dilution and results were classified into four groups: <0.01 IU/mL ; 0.01-0.099 IU/mL ; 0.1-1.0 IU/mL ; >1.0 IU/mL, according to the manufacturer's recommendation. The three last concentration groups (>= 0.01 IU/mL) were considered to be protective against infection [[1]](https://paperpile.com/c/vuVPt1/bGbd).


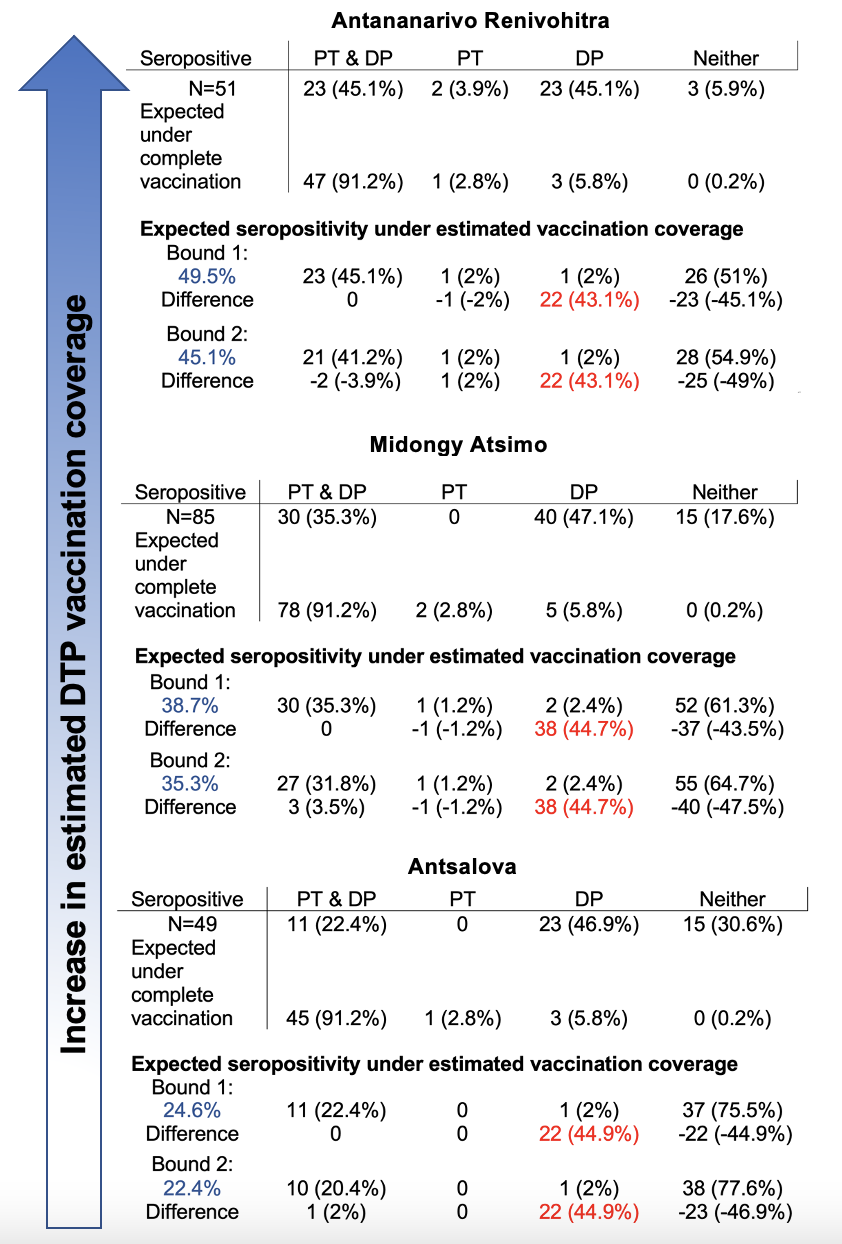


**Supplementary Table S1: Diphtheria and pertussis seropositivity and estimated vaccination coverage among children aged 6-11 months old broken down by district** Diphtheria (DP) and pertussis (PT) seropositivity in children aged 6-11 months in the three districts for which data was available; excess number of diphtheria only seropositive individuals; expectations given vaccine efficacy under complete vaccination and estimated vaccination coverage.

|  |  |  |  |  |
| --- | --- | --- | --- | --- |
|  | 8-15 year old | | | |
| Seropositive | PT & DP | PT | DP | Neither |
| ANT (N=74) | 28 (37.8%) | 17 (23%) | 21 (28.4%) | 8 (10.8%) |
| ATS (N=67) | 51 (76.1%) | 2 (3%) | 13 (19.4%) | 1 (1.5%) |
| MAH (N=81) | 46 (56.8%) | 6 (7.4%) | 29 (35.8%) | 0 |
| MID (N=64) | 45 (70.3%) | 4 (6.3%) | 15 (23.4%) | 0 |
| TOL (N=76) | 45 (59.2%) | 2 (2.6%) | 28 (36.8%) | 1 (1.3%) |
| Total (N=362) | 215 (59.4%) | 31 (8.7%) | 106 (29.3%) | 10 (2.8%) |
| Expected under complete vaccination (N=362) | 330 (91.2%) | 10 (2.8%) | 21 (5.8%) | 1 (0.28%) |

**Supplementary Table S2: Diphtheria and pertussis seropositivity among children aged 8-15 years old** Diphtheria (DP) and pertussis (PT) seropositivity in children aged 8-15 years old in the five districts for which data was available and expectations given vaccine efficacy under complete vaccination. ANT, Antananarivo Renivohitra; ATS, Antsalova; MAH, Mahajanga I; MID, Midongy Atsimo; TOL, Toliara I

|  | 6-11 month old | | | |
| --- | --- | --- | --- | --- |
| Seropositive | PT & DP | PT | DP | Neither |
| ANT (N=51) | 5 (9.8%) | 0 | 41 (80.4%) | 5 (9.9%) |
| ATS (N=49) | 1 (2.0%) | 0 | 33 (67.3%) | 15 (30.6%) |
| MID (N=85) | 3 (3.5%) | 0 | 67 (78.8%) | 15 (17.6%) |
| Total(N=185) | 9 (4.9%) | 0 | 141 (76.2%) | 35 (18.9%) |
| Expected under complete vaccination (N=185) | 169 (91.1%) | 5 (2.8%) | 11 (5.8%) | 0 (0.18%) |
| Expected seropositivity under estimated vaccination coverage |  |  |  |  |
| Bound 1: 9.9% | 9 (4.9%) | 0 | 1 (0.5%) | 175 (94.6%) |
| Difference | 0 | 0 | 140 (75.7%) | -140 (-75.7%) |
| Bound 2: 4.9% | 8 (4.3%) | 0 | 1 (0.5%) | 176 (95.1%) |
| Difference | 1 (0.5%) | 0 | 140 (75.7%) | -141 (-76.2%) |

**Supplementary Table S3: Diphtheria and pertussis seropositivity and estimated vaccination coverage among children aged 6-11 months old** Diphtheria (DP) and pertussis (PT) seropositivity in children aged 6-11 months in the three districts for which data was available; excess number of diphtheria only seropositive individuals; expectations given vaccine efficacy under complete vaccination and estimated vaccination coverage. The threshold for pertussis seropositivity was raised to 40 IU/mL. ANT, Antananarivo Renivohitra ; ATS, Antsalova ; MID, Midongy Atsimo

|  |  |  |  |  |
| --- | --- | --- | --- | --- |
|  | 8-15 year old | | | |
| Seropositive | PT & DP | PT | DP | Neither |
| ANT (N=74) | 4 (5.4%) | 7 (9.5%) | 45 (60.8%) | 18 (24.3%) |
| ATS (N=67) | 14 (20.9%) | 2 (3.0%) | 50 (74.6%) | 1 (1.5%) |
| MAH (N=81) | 6 (7.4%) | 2 (2.5%) | 69 (85.2%) | 4 (4.9%) |
| MID (N=64) | 15 (23.4%) | 1 (1.6%) | 45 (70.3%) | 3 (4.7%) |
| TOL (N=76) | 9 (11.8%) | 1 (1.3%) | 64 (84.2%) | 2 (2.6%) |
| Total (N=362) | 48 (13.3%) | 13 (3.6%) | 273 (75.4%) | 28 (7.7%) |
| Expected under complete vaccination (N=362) | 330 (91.2%) | 10 (2.8%) | 21 (5.8%) | 1 (0.28%) |

**Supplementary Table S4: Diphtheria and pertussis seropositivity among children aged 8-15 years old** Diphtheria (DP) and pertussis (PT) seropositivity in children aged 8-15 years old in the five districts for which data was available and expectations given vaccine efficacy under complete vaccination. The threshold for pertussis seropositivity was raised to 40 IU/mL. ANT, Antananarivo Renivohitra; ATS, Antsalova; MAH, Mahajanga I; MID, Midongy Atsimo; TOL, Toliara I

Supplementary References

1. **Burkovski A**. *Corynebacterium diphtheriae and Related Toxigenic Species: Genomics, Pathogenicity and Applications*. Springer Science & Business Media, 2013.
