# Supplementary figures and images for: Leveraging serology to titrate immunisation programme functionality for diphtheria in Madagascar
Source: Epidemiol Infect. 2022 Jan 13;150:e39. doi: 10.1017/S0950268822000097 (PMC8888278; doi:10.1017/S0950268822000097)

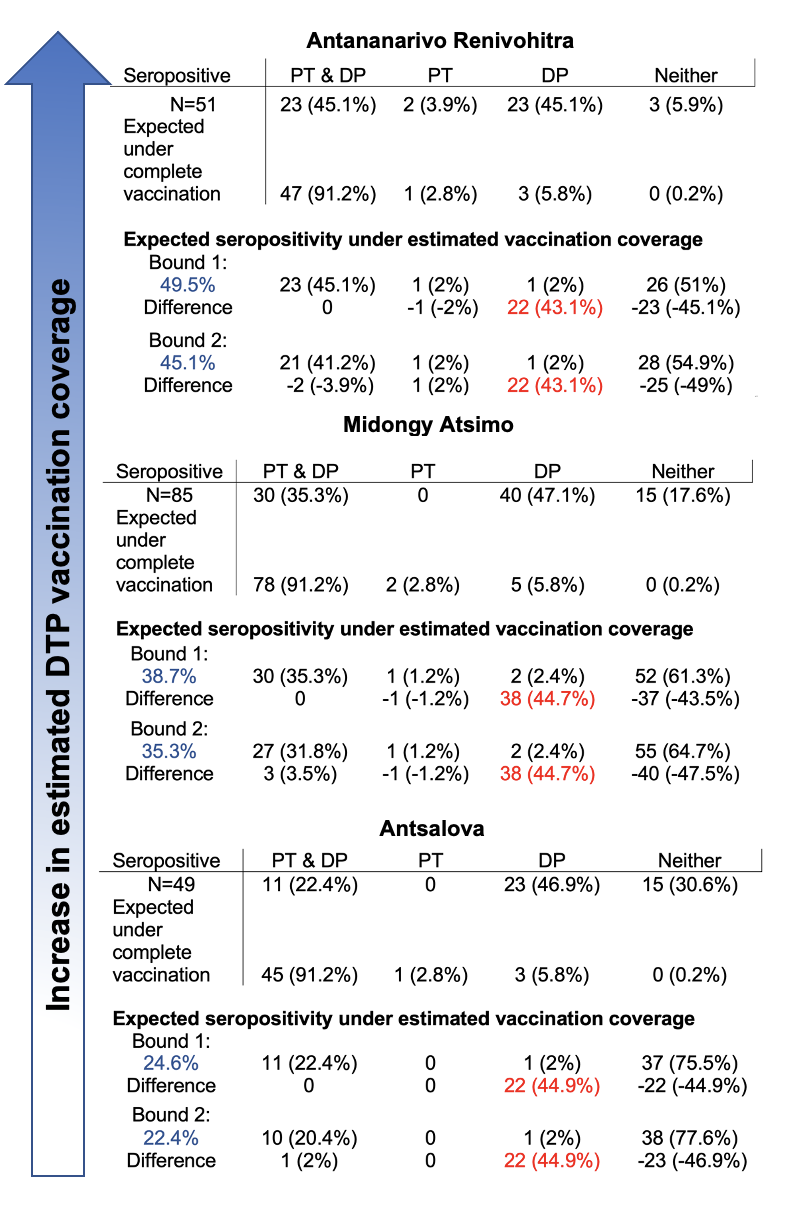

Supplement: Supplementary file 1 [file hygsup.zip › S0950268822000097sup001.tif]
